# Balancing AhR-Dependent Pro-Oxidant and Nrf2-Responsive Anti-Oxidant Pathways in Age-Related Retinopathy: Is SERPINE1 Expression a Therapeutic Target in Disease Onset and Progression?

**DOI:** 10.4172/1747-0862.1000101

**Published:** 2014-07-20

**Authors:** Paul J. Higgins

**Affiliations:** Center for Cell Biology & Cancer Research, Albany Medical College, Albany, New York 12208

## Editorial

**Figure 1:**
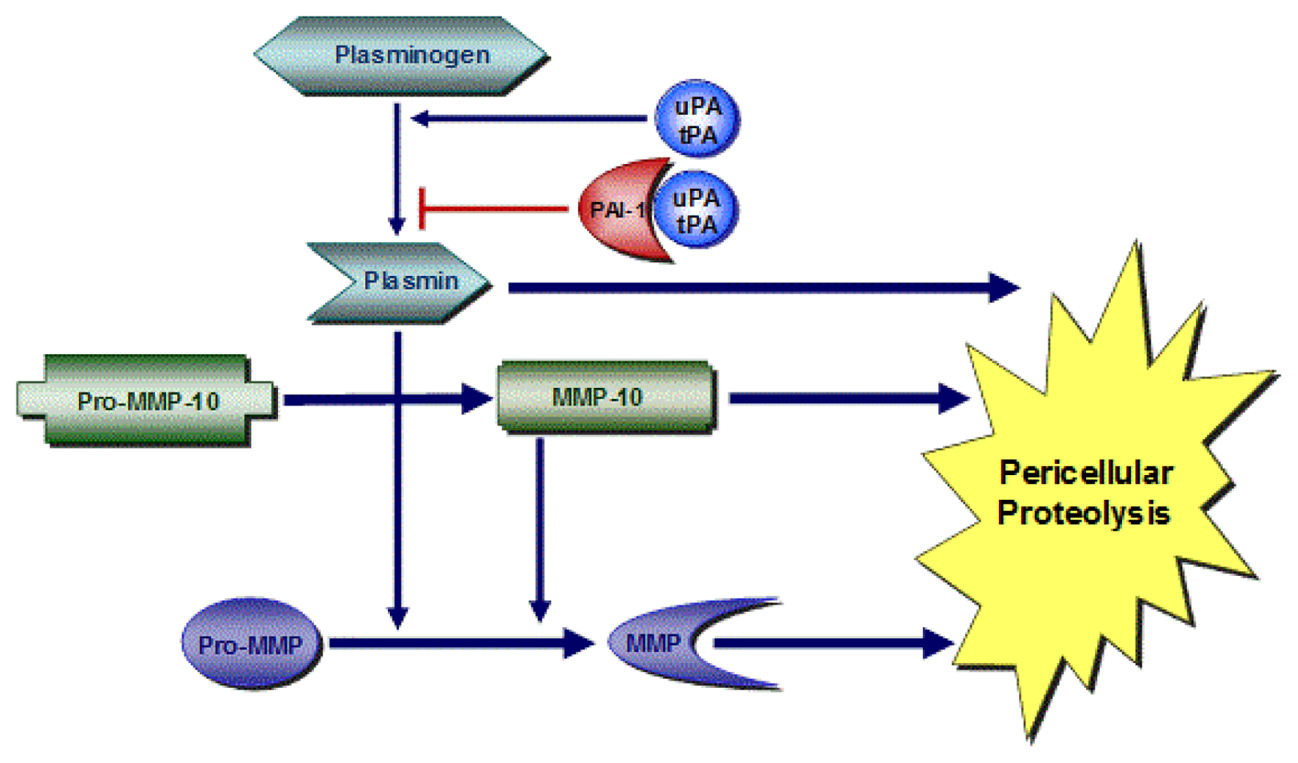
Regulation of the proteolytic microenvironment. A highly-interactive plasmin-matrix metalloproteinase (MMP) pericellular proteolytic cascade is finely “titrated” both temporally and spatially by SERPINE1 (PAI-1). This complex cooperating system of proteases and inhibitors is fundamental to normal tissue repair and development of chronic diseases (adapted from [11]).

Mechanisms underlying the pathophysiology of age-related macular degeneration (AMD), a progressive disease of the retina and a major cause of vision impairment in the elderly, are areas of intense clinical interest. Early-onset AMD is one of the leading causes of blindness in the western world and appears to involve pathologic angiogenesis, unresolving inflammation, oxidative stress and choroidal fibrosis. It is likely that the development of AMD is largely multifactorial involving chronic exposure to xenobiotics, genetic predisposition to initiation and progression, generation of free radicals and an insufficient anti-oxidant defense system. In this issue of the *Journal*, Perepechaeva et al. [1] explore the dysregulation of AhR-Nrf2 “gene batteries” in the retinas of senescence-accelerated OXYS rats during the emergence of an AMD-like retinopathology. The impetus for this work appears to stem from the recent inclusion of genes that encode the arylhydrocarbon receptor (AhR) and nuclear factor erythroid 2-related factor 2 (Nrf2), transcription factors essential to the regulation of the global cellular oxidant control program, among the repertoire of candidates that may be involved in either the predisposition to, or development of, AMD. Perepechaeva et al. [1] discuss the biology underlying AhR regulation of the expression of several cytochrome P450 genes, as well as others, involved in xenobiotic biotransformation and, thereby, the generation of oxidative stress. Nrf2, in contrast, is an oxidative stress-responsive transcription factor that controls an antioxidant response. The intriguing finding is that AhR mRNA was reduced in the retinas of senescence-accelerated AMD-prone OXYS rats while Nrf2 transcripts were increased, although other critical AhR/Nrf2-dependent genes were decreased. One may reasonably speculate that changes in the expression of a subset of AhR/Nrf2 genomic targets may initiate oxidative stress associated with dysregulation of an AMD “gene battery”, triggered perhaps by reduced AhR levels. Indeed, Perepechaeva et al. [1] suggest that oxidative stress may be induced by, or develop as a consequence, of malfunction in the balance between the reactive oxygen species (ROS)-generating and anti-oxidant pathways. Since AhR is required for a number of physiologic processes and AhR-deficient mice have a hyper-inflammatory phenotype, decreased AhR may contribute to a prolonged oxidative stress. These findings highlight previous studies on the role of specific genes in retinal disease also related to the stress phenotype. Earlier investigations reported high levels of plasminogen activator inhibitor-1 (PAI-1, SERPINE1), a serine protease inhibitor and the major negative regulator of the plasmin-based pericellular proteolytic cascade, in AMD (Figure 1). SERPINE1 has been causally implicated in tissue fibrosis and pathologic angiogenesis, and in neovascularization characteristic of ocular pathologies including diabetic retinopathy and AMD [2,3]. This has mechanistic significance to the pathology of AMD which is characterized by thickening of Bruch’s membrane due to accumulation of extracellular matrix (ECM). The role of ROS in AMD pathophysiology is highlighted by the induction of the profibrotic factors SERPINE1 and connective tissue growth factor (CTGF) in hypoxia/reoxygenation in cultured human retinal pigment epithelial cells and the attenuation of this response by antioxidants [4]. SERPINE1 is significantly elevated in the aqueous humor of patients with diabetic macular edema [5]. The available data highlight a proangiogenic role for SERPINE1 in pathogenic choroidal neovascularization and suggest that targeting SERPINE1 expression and/or function may have translational implications as an AMD therapy [2,3]. Indeed, the SERPINE1 gene is induced in response to elevated ROS in response to TGF-β1, a mechanism that involves EGFR^Y845^ transactivation and generation of p53/SMAD3 transcriptional complexes [6–8]. While the responding promoter sequences appear different between AhR-dependent and TGF-β1-mediated transcription, AhR-directed SERPINE1 induction is evident in wild-type cells but not in AhR-null mutants and α-naphthoflavone and phenanthroline, two AhR antagonists, inhibit SERPINE1 promoter-driven reporter gene expression in response to the AhR activator TCDD [9].

**Figure 2:**
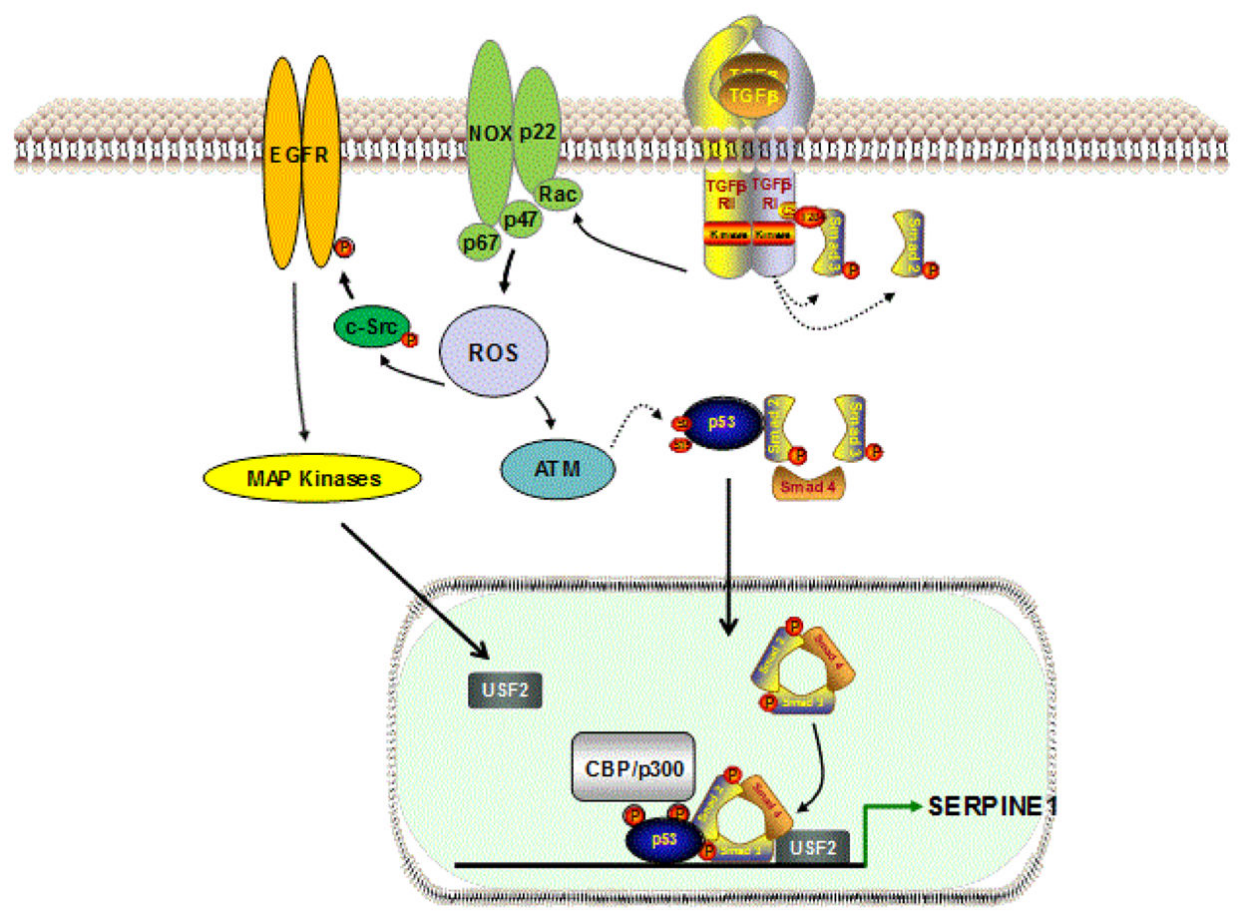
A model for ROS involvement in SERPINE1 induction by TGF-β1. TGF-β1 receptor activation upon ligand binding activates Smad 2/3 (via phosphorylation by the ALK5/TGF-β1 receptor 1 type) as well as non-Smad (e.g. EGFR, MAPK, Akt, Rho-ROCK) signaling cascades with downstream effects on gene expression (e.g. SERPINE1). Rapid ROS generation in response to TGF-β1 stimulation appears critical for initiation of non-SMAD (e.g. EGFR, SRC) pathways. p53 integrates transcriptional contributions from both SMAD and non-SMAD cascades, as well as with accessory cofactors (e.g., USF2), to attain maximal SERPINE1 expression (modified from [15]).

Knock down of Nrf2, in contrast, significantly increased SERPINE1 expression consistent with its implication as a SERPINE1 repressor [10]. The actual role, if any, of TGF-β isoforms in AMD is unclear [12,13]. However, TGF-β1 stimulates NOX-dependent ROS generation and activation of the p53-SMAD2/3 pathways leading to SERPINE1 induction [6–8] (Figure 2). Multivariate regression revealed a significant relationship between urinary TGF-β1 levels and AMD (odds ratio 1.24; P<0.031) [14]. Elegant animal studies, in fact, confirmed that SERPINE1 was required for choroidal neovascularization in a laser-induced injury model that shares certain features with AMD [2,3]. As suggested by Perepechaeva et al. [1] in this issue, dysregulation of the finely-tuned balance between AhR and Nrf2 response genes (including SERPINE1 as well as CYPA1, CYPA2, NQO1, UGT1A6, GSTA1) may contribute to the eventual onset and progression of choroidal neovascularization and associated retinopathies.
